# Antimicrobial effects of nanocurcumin gel on reducing the microbial count of gingival fluids of implant‒abutment interface: A clinical study

**DOI:** 10.34172/japid.2022.014

**Published:** 2022-08-27

**Authors:** Mohammad Ali Ghavimi, Shahriar Shahi, Solmaz Maleki Dizaj, Simin Sharifi, Ali Noie Alamdari, Amir Reza Jamei Khosroshahi, Khadijeh Khezri

**Affiliations:** ^1^Department of Oral and Maxillofacial Surgery, Faculty of Dentistry, Tabriz University of Medical Sciences, Tabriz, Iran; ^2^Dental and Periodontal Research Center, Tabriz University of Medical Sciences, Tabriz, Iran; ^3^Department of Pediatric Dentistry, Faculty of Dentistry, Tabriz University of Medical Sciences, Tabriz, Iran; ^4^Deputy of Food and Drug Administration, Urmia University of Medical Sciences, Urmia, Iran; ^5^Department of Nursing, Khoy University of Medical Sciences, Khoy, Iran

**Keywords:** Antimicrobial agents, curcumin, dental implant, nanogel

## Abstract

**Background.** This clinical study aimed to prepare and evaluate the effect of antimicrobial nanocurcumin gel on reducing the microbial counts of gingival fluids of the implant‒abutment interface in patients referred to the Tabriz Faculty of Dentistry for the placement of two dental implants.

**Methods.** Fifteen patients applying for at least two dental implants were included in the study. During the uncovering session, nanocurcumin gel was placed in one implant, and no substance was placed in another (the control group). Then, in three sessions, implantation sessions (10 days after the repair abutment closure session), prosthesis delivery (15 days after the implantation session), and one month after prosthesis delivery, the patients’ gingival fluid was sampled and cultured to determine bacterial counts in the gingival fluid by colony-forming units (CFU/mL). T-test was used for statistical analysis of data, and statistical significance was set at P<0.05.

**Results.** This study showed that nanocurcumin gel significantly reduced the CFU/mL of gingival fluid in all three sampling stages compared to the control group.

**Conclusion.** According to the results of this study, the application of antimicrobial nanocurcumin gel inside the implant fixture could reduce the microbial counts of gingival fluids.

## Introduction

 Patients and dentists have always considered implant treatment as a replacement for missing teeth. Implants are attached to the jawbone through osseointegration. According to clinical reports, the complete osseointegration process takes 2‒4 months.^1,2^ Over the years, it has been demonstrated that implant treatment sometimes fails. Infection is one of the main reasons for the failure of implant treatment. One of the most common infections and gingival diseases is periodontal disease, caused by poor oral hygiene and attached plaque on tooth surfaces.^3^

 Among capnophilic bacteria, *Aggregatibacter actinomycetemcomitans, Eikenella corrodens, *and *capnocytophaga*, and anaerobic bacteria *Porphyromonas gingivalis, Prevotella intermedia, Fusobacterium nucleatum, Tannerella forsythia, Treponema denticola,* and *Actinomyces spices* play a key role in periodontal disease.^4-11^

 Bacterial colonization AT the implant‒abutment interface depends on three factors: implant system, dynamic loading, and preventive treatment in the interface area using disinfectants and sealing materials.^12-15^

 In a report by Podhorsky et al,^15^ two types of sealants (Berutemp 500 T2, Kiero seal) and two types of disinfectants (1% CHX gel and GlaxoSmithKline) reduced bacterial colonization at the interface but did not remove the bacteria completely. Ferrari et al^16^ showed that disinfection of the implant‒abutment interface with iodine solution could not prevent re-colonization of future periodontal pathogens.

 Numerous side effects of synthetic drugs and drug resistance have led to a tendency to natural-origin antimicrobial agents such as plants. Curcumin (Diferuloylmethane) is the active component of turmeric and has very strong antimicrobial and anti-inflammatory properties. This substance has been approved by FDA. Recent studies have shown that antibacterial substances made of curcumin against *Staphylococcus epidermidis, Staphylococcus aureus, Klebsiella pneumoniae, and Escherichia coli* have 16-32 minimum bacterial concentration (MBC) and 4-16 minimum inhibitory concentration (MIC).^17,18^ Also, recent studies have shown that the methanolic extract of turmeric has a 128-μg/mL MIC against *S. aureus*.^19^ Also, a mixture of curcumin extract with other antibacterial substances has been used to produce gels and facial emulsions to improve skin care and wound coverage.^20^

 Nandini et al^21^ compared the effectiveness of the 1% curcumin solution with 2.5% chlorhexidine solution in the control of chronic periodontitis. In that research, patients were divided into three groups: curcumin recipients, chlorhexidine recipients, and 0.9% saline recipients. The patients were evaluated for 15 days and 1 month after receiving the intervention. The results showed that 1% curcumin solution was as effective as a 0.2% chlorhexidine mouthwash. The effect of curcumin on *S. mutans* on tooth surfaces and extracellular matrix proteins was also investigated in another study. The results showed that the MIC for the complete inhibition of *S. mutans* attached to human teeth was 128 μg/mL.^22^

 Sha et al^23^ showed that *P. gingivalis* isolated from patients is highly sensitive to curcumin at a low MIC of 12.5 μg/mL.

 Considering the antibacterial effects of curcumin in recent studies and following our two previous in vitro studies,^24,25^ and since infection is one of the reasons for implant failures, this study aimed to prepare and evaluate antimicrobial nanocurcumin gel for reducing the microbial infection at the implant‒abutment interface clinically.

## Methods

###  Target population 

 The current study was a clinical trial on patients referred to the Tabriz Faculty of Dentistry, requiring at least two dental implants.

###  Inclusion criteria

- Patients diagnosed with moderate to high levels of oral hygiene
- Having at least two implants in one jaw
- At least 18 years old
- 1-3 mm of probing depth around the implant
- Zirconia crown with supragingival margins^26^

###  Exclusion criteria

 Patients diagnosed with low or very low levels of oral hygiene

 Pregnant or lactating women

 Patients who had received antibiotics in the past six months

 Patients with any systemic disease such as diabetes, affecting oral health

 Addiction to drugs, cigarettes, or alcohol^26^.

###  Sample size determination

 Fifteen samples were included based on the results of a study by Mombelli et al^26^ and considering a type 1 error rate of 5% and a test power of 80%.

###  Nanocurcumin gel preparation

 Curcumin nanocrystals (Alborz, Tehran, Iran) with a mean particle size of 80 nm were suspended in distilled water (1% w/w). Then, 2% w/w carbomer 940 (MilliporeSigma, Germany) was added to the suspension and mixed gently until the gel formed.

###  Microbial test

 According to the defined inclusion and exclusion criteria, 15 patients referred to the Tabriz Faculty of Dentistry, requiring at least two implants in one jaw (Osstem implant system [length=11.5 and diameter=4 mm]) were included in the study. In the first session, nanocurcumin gel was placed inside the implant screw (test), no substance was used in another implant (control), and a healing abutment was placed. Then, the samples were collected from the patients in three sessions, including impression (10 days after the healing abutment placement session), prosthesis insertion (15 days after the impression session), and one month after prosthesis insertion. Gingival fluid was used for sampling. Each area was isolated with a cotton roll and air-dried for 5 minutes to remove saliva from the area. The samples were collected using paper points inserted into the gingival sulcus in four distinct areas (mesiobuccal, distobuccal, mesiolingual, and distolingual) to the extent that moderate resistance was felt and held there for 30 seconds. Paper points with blood contamination were discarded. After sampling, each paper cone was placed in small sterile tubes, and the prepared samples were sent to the microbiology laboratory to determine bacterial counts.

 Before the culture procedures, the transfer medium was vortexed for 30 seconds. In the next stage, culture was performed on the desired media with standard loops. Brucella agar medium containing 5% defibrinated sheep blood was used as a culture medium, and horse serum (5%) and vitamin K1 were used to enrich the culture medium. After 72 hours of incubation at 37°C, bacterial counts in the gingival fluid were determined using the counting method.

###  Data analysis 

 T-test was used for the statistical analysis of data. Statistical significance was set at P<0.05.

## Results

 The results of this study showed that in all three stages of sampling, nanocurcumin gel significantly reduced the CFU/mL of gingival fluid compared to the control group. The results of the CFU/mL in the studied groups are presented in Table 1.

**Table 1:** The results of the CFU/mL in the studied groups

**Groups**	**First session**		**Second session**		**Third session**	
	**Mean**	**SD**	**Mean**	**SD**	**Mean**	**SD**
**Gel**	599.79	85.16	6420.10	481.65	31001.00	11102.21
**Control**	1411787.30	969.09	4042458.22	817380.90	39994568.03	134962.77
**P-value**	<0.0001		0.0022		<0.0001	

 The results also showed that the percentage of inhibition (inhibition rate [IR%]) in terms of the gel in all three stages (sessions) was >99%. The growth inhibition rate of the gel group is presented in Table 2.

**Table 2:** Growth inhibition rate of gel group

**Sessions**	**Control (CFU/mL)**	**Log CFU/mL Control**	**Gel (CFU/mL)**	**Log CFU/mL gel**	Gel IR*
**1**	1411787.30	6.14	599.79	2.77	99.95
**2**	4042458.22	6.60	6420.10	3.80	99.84
**3**	39994568.03	7.60	31001.00	4.49	99.92

IR*= (CFU/mL of control – CFU/mL of gel)/ CFU/mL of control×100

## Discussion

 Failure in implant treatments can be attributed to biological, mechanical, iatrogenic, or functional factors that lead to bacterial infections of peri-implant tissues and implant overloading.^26^ Identifying these factors affects the success of implant treatments. In many cases, the cause of these failures remains unknown. Therefore, researchers are always looking for failure factors or complications of implant treatments. The occurrence of infections after implant placement is one of the main reasons for the failure of implant treatment, and failed treatments are associated with a type of microbial flora that is directly related to periodontitis.^27,28^ Peri-implantitis is a destructive inflammatory disease that can cause the loss of dental implants,^29^ and clinical studies have shown that the highest incidence occurs in the first 12 months (early failure) after implant placement.^11,30^ If this disease occurs, it is treated by mechanical debridement with chemical antiseptics.^30^

 The advantages of using low concentrations of antibiotics include reducing cytotoxic effects and bacterial resistance.^31^ Our findings showed that in all sampling stages, the gel group significantly reduced CFU more than the control group.

 Our results showed IR values of >99% in all three sessions for the gel group. In a previous study of our group, Negahdari et al^24^ studied the effects of curcumin (60 mg/mL), chlorhexidine, and water on *E. coli*,* E. faecalis*,and* S. aureus* in implant fixtures in vitro. The implants were incubated at 37°C for 24, 48, and 72 hours. The contents of each implant were cultured for 24 hours to count bacterial colonies at 37°C. The results showed that curcumin eliminated 99.99% of all bacteria. In addition, the colony-forming unit (CFU) of bacteria exposed to curcumin decreased significantly over time (P<0.01). In another study of this group, we examined the effect of the increasing amount of torque on healing abutment. In that study, the effect of nanocurcumin (60 mg/mL), chlorhexidine, and water on *E. coli, E. faecalis*, and *S. aureus* were studied in vitro on an implant fixture. The implants were incubated at 37°C for 24 hours by applying three different torques of 10, 20, and 35. The contents of each implant were cultured for 24 hours to count bacterial colonies at 37°C. The results showed that curcumin nanocrystals eliminated 99.99% of all bacteria. The results also showed that by increasing the applied torque, the CFU level decreased significantly (P<0.01).^25^

 Previous in vitro studies of this group showed the role of curcumin in reducing bacterial colonies, which was performed to evaluate the antimicrobial effect of curcumin and select the appropriate torque. The present study used the nanocurcumin gel clinically. This study showed that in all three stages, nanocurcumin gel significantly reduced the CFU/mL of the gingival fluid compared to the control group. Due to the presence of patients, access to the oral microbial flora was possible. Scientific sources show that bacterial species isolated from patients lead to different results compared to standard laboratory species. Laboratory species kept in microbial banks are less pathogenic or non-pathogenic.^23,26^ In addition, this method can be used to evaluate the frequency of periodontopathogenic bacteria in patients. The microbial culture method can be used to estimate the percentage of infections caused by capnophilic bacteria and anaerobic bacteria or due to both cases in the patient population.^23^

 Previous investigations also showed that the local application of curcumin gel reduced gingival inflammation.^32-34^ In addition, there was evidence that curcumin efficiently inhibits the inflammatory mediators’ activation and has therapeutic influences on periodontal diseases.^32,33^ Also, Cirano et al^35^ reported that curcumin enhanced bone volume and improved bone‒implant interactions in an animal model

 The interaction of nanomaterials with the bacterial membrane creates local holes in the membrane, damaging the bacteria. The nanoparticles-membrane binding gradually results in the penetration of antimicrobial nanoparticles into the bacterial cytoplasm, disturbing bacterial functions.^36,37^

## Conclusions

 Based on the current study, the application of antimicrobial nanocurcumin gel inside the implant fixture can reduce the microbial count of gingival fluids. Herbal nanoparticles can be used in the future to replace chemical antimicrobials or reduce bacterial resistance. Sustained-release formulation of curcumin can also increase its clinical efficacy and improve patient management.

## Acknowledgments

 This article was written based on a dataset from a thesis registered at Tabriz University of Medical Sciences (#65871). The thesis was supported by the Vice Chancellor for Research at Tabriz University of Medical Sciences, which is greatly acknowledged.

## Competing interests

 The authors declare no competing interests in this study.

## Authors’ contributions

 MG, SMD, SS, and SS designed the study. AN, AJ, and KK wrote the initial draft of the manuscript. SMD and SS revised the draft. All authors contributed to the manuscript’s writing and critical revision. All authors read and approved the final version of the manuscript.

## Funding

 This study was funded by the Vice-Chancellor for Research (VCR) of Tabriz University of Medical Sciences.

## Availability of data

 The raw/processed data required to reproduce these findings can be shared after publication by request from the corresponding author.

## Ethics approval

 This study was approved by the Ethics Committee of Tabriz University of Medical Sciences (Ethical code: IR.TBZMED.REC.1399.605). Written informed consent was obtained from all patients.

## References

[1] Desai CT, Desai SJ, Marjadi DS, Shah GS (2018). Diminution of internal bacterial contamination of external dental implants using silver nanoparticles. International Journal of Agricultural Science Research and Technology.

[2] Adell R, Eriksson B, Lekholm U, Brånemark P-I, Jemt T (1990). A long-term follow-up study of osseointegrated implants in the treatment of totally edentulous jaws. International Journal of Oral & Maxillofacial Implants.

[3] Schierholz J, Beuth J (2001). Implant infections: a haven for opportunistic bacteria. Journal of Hospital Infection.

[4] Ogunsalu C, Daisley H, Akpaka P (2011). Prevalence and Antimicrobial Susceptibility Pattern of Pathogens Isolated from Patients with Juvenile Periodontitis in Jamaica: A Prospective Multi-centre Study of 15 Cases over a 15-year Period. West indian medical journal.

[5] Kuramitsu HK, He X, Lux R, Anderson MH, Shi W (2007). Interspecies interactions within oral microbial communities. Microbiology and molecular biology reviews.

[6] Good TJ, Kahook MY (2011). Assessment of bleb morphologic features and postoperative outcomes after Ex-PRESS drainage device implantation versus trabeculectomy. American journal of ophthalmology.

[7] Matthiesen L, Berg G, Ernerudh J, Ekerfelt C, Jonsson Y, Sharma S (2005). Immunology of preeclampsia. Immunology of Pregnancy.

[8] Socransky S (1970). Relationship of bacteria to the etiology of periodontal disease. J dent res.

[9] Franklin PD, Allison JJ, Ayers DC (2012). Beyond joint implant registries: a patient-centered research consortium for comparative effectiveness in total joint replacement. Jama.

[10] Grant B-T, Amenedo C, Freeman K, Kraut RA (2008). Outcomes of placing dental implants in patients taking oral bisphosphonates: a review of 115 cases. Journal of Oral and Maxillofacial Surgery.

[11] Berglundh T, Persson L, Klinge B (2002). A systematic review of the incidence of biological and technical complications in implant dentistry reported in prospective longitudinal studies of at least 5 years. Journal of clinical periodontology.

[12] Scarano A, Assenza B, Piattelli M, Iezzi G, Leghissa GC, Quaranta A (2005). A 16–year study of the microgap between 272 human titanium implants and their abutments. Journal of Oral Implantology.

[13] Wise R, Hart T, Cars O, Streulens M, Helmuth R, Huovinen P, et al. Antimicrobial resistance. British Medical Journal Publishing Group; 1998. p. 609-10. 10.1136/bmj.317.7159.609 PMC1113826 9727981

[14] Mohn D, Zehnder M, Stark WJ, Imfeld T (2011). Electrochemical disinfection of dental implants–a proof of concept. PloS one.

[15] Podhorsky A, Putzier S, Rehmann P, Streckbein P, Domann E, Wöstmann B (2016). Bacterial contamination of the internal cavity of dental implants after application of disinfectant or sealant agents under cyclic loading in vitro. Int J Prosthodont.

[16] Ferrari RB, De Lorenzo JL, Ferrari DS, Shibli JA, Sendyk WR (2009). Microbial recolonization of the internal surfaces of the implant-abutment junction after disinfection with iodine solution: A pilot study. Revista Saúde-UNG-Ser.

[17] Ungphaiboon S, Supavita T, Singchangchai P, Sungkarak S, Rattanasuwan P, Itharat A (2005). Study on antioxidant and antimicrobial activities of turmeric clear liquid soap for wound treatment of HIV patients. Songklanakarin Journal of Science and Technology.

[18] Ammon HP, Wahl MA (1991). Pharmacology of Curcuma longa. Planta medica.

[19] Araujo C, Leon L (2001). Biological activities of Curcuma longa L. Memórias do Instituto Oswaldo Cruz.

[20] Hayakawa H, Minaniya Y, Ito K, Yamamoto Y, Fukuda T (2011). Difference of curcumin content in Curcuma longa L. (Zingiberaceae) caused by hybridization with other Curcuma species. American Journal of Plant Sciences.

[21] Nandini N, Vidya D, Komal A (2012). Comparative evaluation of 1% Curcumin solution and 0.2% Chlorhexidine irrigation as an adjunct to scaling and root planing in management of chronic periodontitis: A clinico-microbiological study. Journal of Pharmaceutical and Biomedical Sciences (JPBMS).

[22] Song J, Choi B, Jin E-J, Yoon Y, Choi K-H (2012). Curcumin suppresses Streptococcus mutans adherence to human tooth surfaces and extracellular matrix proteins. European journal of clinical microbiology & infectious diseases.

[23] Sha AM, Garib BT (2019). Antibacterial effect of curcumin against clinically isolated Porphyromonas gingivalis and connective tissue reactions to curcumin gel in the subcutaneous tissue of rats. BioMed research international.

[24] Negahdari R, Sharifi S, Ghavimi MA, Memar MY, Khaneshi B, Maleki Dizaj S (2020). Curcumin nanocrystals: production, physicochemical assessment, and in vitro evaluation of the antimicrobial effects against bacterial loading of the implant fixture. Applied Sciences.

[25] Negahdari R, Ghavimi MA, Barzegar A, Memar MY, Balazadeh L, Bohlouli S (2021). Antibacterial effect of nanocurcumin inside the implant fixture: An in vitro study. Clinical and Experimental Dental Research.

[26] Mombelli A, Feloutzis A, Brägger U, Lang NP (2001). Treatment of peri-implantitis by local delivery of tetracycline: Clinical, microbiological and radiological results. Clinical oral implants research.

[27] Alcoforado G, Rams T, Feik D, Slots J (1991). Microbial aspects of failing osseointegrated dental implants in humans. Journal de parodontologie.

[28] Mombelli A, Van Oosten M, Schürch Jr E, Lang N (1987). The microbiota associated with successful or failing osseointegrated titanium implants. Oral microbiology and immunology.

[29] Mombelli A (2002). Microbiology and antimicrobial therapy of peri-implantitis. Periodontology 2000.

[30] Snauwaert K, Duyck J, van Steenberghe D, Quirynen M, Naert I (2000). Time dependent failure rate and marginal bone loss of implant supported prostheses: a 15-year follow-up study. Clinical oral investigations.

[31] Shahi R, Albuquerque M, Münchow E, Blanchard S, Gregory R, Bottino M (2017). Novel bioactive tetracycline-containing electrospun polymer fibers as a potential antibacterial dental implant coating. Odontology.

[32] Farjana HN, Chandrasekaran S, Gita B (2014). Effect of oral curcuma gel in gingivitis management-a pilot study. Journal of clinical and diagnostic research: JCDR.

[33] Guimaraes-Stabili MR, de Aquino SG, de Almeida Curylofo F, Tasso CO, Rocha FRG, de Medeiros MC (2019). Systemic administration of curcumin or piperine enhances the periodontal repair: a preliminary study in rats. Clinical oral investigations.

[34] Malekzadeh M, Kia SJ, Mashaei L, Moosavi MS (2021). Oral n ano-curcumin on gingival inflammation in patients with gingivitis and mild periodontitis. Clinical and experimental dental research.

[35] Cirano FR, Pimentel SP, Casati MZ, Corrêa MG, Pino DS, Messora MR (2018). Effect of curcumin on bone tissue in the diabetic rat: repair of peri-implant and critical-sized defects. Int J Oral Maxillofac Surg.

[36] Salatin S, Maleki Dizaj S, Yari Khosroushahi A (2015). Effect of the surface modification, size, and shape on cellular uptake of nanoparticles. Cell biology international.

[37] Wang L, Hu C, Shao L (2017). The antimicrobial activity of nanoparticles: present situation and prospects for the future. International journal of nanomedicine.

